# Crossbreeding beef sires to dairy cows: cow, feedlot, and carcass performance

**DOI:** 10.1093/tas/txac059

**Published:** 2022-05-09

**Authors:** Blake A Foraker, Michael A Ballou, Dale R Woerner

**Affiliations:** Department of Animal and Food Sciences, Texas Tech University, Lubbock, TX 79409, USA; Department of Veterinary Sciences, Texas Tech University, Lubbock, TX 79409, USA; Department of Animal and Food Sciences, Texas Tech University, Lubbock, TX 79409, USA

**Keywords:** Beef on Dairy, feed efficiency, lactation performance, quality grade, yield grade

## Abstract

Genetic and reproductive advancements in the dairy industry, volatile milk markets, and beef packer restrictions on dairy carcasses have increased the popularity of crossbreeding beef sires to dairy cows in the United States. This observational study aimed to understand performance of dairy cows bred to beef sires and feedlot and carcass performance of crossbred beef × dairy cattle. For dairy cow performance, archived records from two dairies representing two successive lactations were evaluated in cows (Dairy A: *n* = 72/group; Dairy B: *n* = 456/group) representing 1) All Dairy, where previous sire type of conception was Holstein for both lactations, or 2) Beef on Dairy, where previous sire type of conception was Holstein for the preceding lactation and a beef breed for the subsequent lactation. For feedlot performance, closeout data from pens (*n* = 26/cattle type) of beef and beef × dairy steers and heifers were evaluated. For carcass performance, individual carcass data were compared between conventional beef (*n* = 966), beef × dairy (*n* = 518), and Holstein (*n* = 935) steers sampled across a variety of processing facilities, harvest lots, and geographical regions. Cow lactation performance was minimally impacted by sire type of previous conception. Cows conceived to beef sires exhibited a 2 to 3 d greater (*P* < 0.01) gestation length than cows conceived to Holstein sires. Beef × dairy cattle were not largely different in weight gain at the feedlot but exhibited 1-unit lesser (*P* < 0.01) dressing percentage than beef cattle. Beef × dairy carcasses possessed 18% lesser (*P* < 0.05) 12th rib fat thickness than beef cattle and 5% greater (*P*< 0.05) ribeye area than dairy cattle. Additionally, beef cattle produced nearly double (*P* < 0.05) the percentage of yield grade 4 carcasses produced by beef × dairy and Holstein cattle.

## INTRODUCTION

Development and success of sexed semen technology and genomic selection have led many U.S. dairies to take a more strategic approach in mating decisions for retained heifer ownership. Meanwhile, the U.S. milk market has demonstrated volatility and created economic hardships for dairy farmers. Recent rejection of Holstein steers at major U.S. beef packers has not helped matters, as it prompted an estimated $610 million revenue loss to U.S. Holstein feeding operations in 2017 (McKendree et al., 2020). These phenomena, coupled with greater value of crossbred beef × dairy calves compared to straightbred dairy calves (McCabe et al., 2022), have encouraged many dairy farmers to impregnate dairy cows with semen with beef sires, a practice commonly labeled “Beef on Dairy.” Rapid and widespread adoption of this breeding practice has prompted many unanswered questions through the beef and dairy supply chains. Dairy producers may be deterred from long-term acceptance of the practice if it negatively influences cow and progeny performance.

Irish studies in the 1980s previously reported that breed of calf sire, whether dairy or beef, had no adverse effect on milk production and minimal effect on reproductive traits in dairy cows (Badi et al., 1985; O’Ferrall and Ryan, 1990). Still, the only recent U.S. study to report on this effect (Scanavez and Mendonça, 2018) concluded that sire breed affected gestation length and produced mixed results on milk yield, depending on the breed of the dam (Holstein vs. crossbred). McWhorter et al. (2020) concluded that conception rate was not largely different between Holstein and Angus sires mated to Holstein cows and that the practice of beef × dairy crossbreeding could be used to produce more valuable, terminally bound calves. Feedlot growth and carcass performance of conventional beef and Holstein steers have been extensively studied, particularly related to the use of beta-adrenergic agonists (Rust and Abney, 2005; Duff and McMurphy, 2007; Beckett et al., 2009; Arp et al., 2014). Baisel and Felix (2022) comprehensively reviewed literature and concluded that most research on feedlot and carcass performance of beef × dairy crossbreds has occurred outside of the United States, where genetics and management practices differ greatly from those in the United States. This observational study aimed to provide an understanding of performance in dairy cows bred to beef sires and provide largely absent feedlot and carcass performance data for beef × dairy crossbreds in the U.S.

## MATERIALS AND METHODS

Data for this study were collected in the field at cooperating producers or federally inspected harvest facilities. Thus, Institutional Animal Care and Use Committee approval was not required.

### Dairy Cow Performance

Archived cow files from Dairy Comp 305 (Valley Agricultural Software, Tulare, CA) were obtained from two commercial dairies: Dairy A, three locations in southwest Kansas and northwest Oklahoma, and Dairy B, one location in northwest Colorado. Each location of both dairies contained at least 5,000 cows. These large-scale dairies were selected for their extensive use of conventional (unsexed) beef semen alongside conventional dairy semen. Each dairy differed in its selection of cows to breed to beef semen; thus, the dairies were analyzed separately. Dairy A made no distinction based on productivity level between cows bred to dairy or beef semen, while Dairy B selectively bred higher-producing cows to dairy semen and lower-producing cows to beef semen. A consultant version of Dairy Comp 305 software was used to extract identity and performance data: animal identity, cow breed, lactation number, previous conception date, previous conception sire, previous days open, previous number of times bred, gestation length, calving interval, days in milk at first breeding, days open, total milk yield, total days in milk, 305-d mature herd equivalent (MHE), peak daily milk yield, average daily milk yield in week 4 (Dairy A only), days in milk at peak production, days dry prior to freshening, stillbirth incidence, twinning incidence, calf sex, laminitis incidence, and mastitis incidence. Birth weight data were not available.

Data were processed and analyzed using R statistical software (R Core Team, 2021). Cases that contained missing data, a previous conception date before 2014, or both were removed. Only multiparous cows with two or more complete lactation records were selected. Data across two successive lactations were identified for each cow, such that some cows with more than two lactation records contained multiple pairs of successive lactations. This filtering process was done because lactation performance of primiparous cows has been shown drastically different from multiparous cows, which were more consistent from lactation to lactation (Mellado et al., 2011). Cows were then classified into two groups: 1) All Dairy—where previous sire type of conception was Holstein for both lactations, or 2) Beef on Dairy—where previous sire type of conception was Holstein for the preceding lactation (I) and a beef breed for the subsequent lactation (II). Other breeding strategies, such as conception to only a beef sire or conception to a beef sire then Holstein sire across successive lactations, were not evaluated because adequate data were not available. Moreover, these strategies were not reflective of progressive mating schemes in most U.S. dairies. In other words, when dairies have interest in heifer calf retention, virgin heifers and younger cows are rarely mated to beef sires because they generally represent the most genetically advanced portion of the herd.

The MatchIt (Ho et al., 2011) and optmatch (Hansen and Klopfer, 2006) packages were used to select control cow records (All Dairy) that matched treatment cow records (Beef on Dairy) in a 1:1 ratio, without replacement, according to criteria: same dairy and location, same cow breed, same lactation number for both lactations, and same season and year of previous conception for both lactations. Seasons were defined as spring (March to May), summer (June to August), fall (September to November), and winter (December to February), according to reasonable climatic patterns for the geographical region. Factors used as matching criteria have been shown to impact cow performance (McDowell et al., 1975; McAllister, 2002; Mellado et al., 2011).

Characteristics of cows meeting selection criteria for both dairies are presented in Table 1. Matched records were returned for 72 cows in each group at Dairy A and 456 cows in each group at Dairy B. Cows were not matched on individual sire of previous conception so that a variety of different individual sires were represented. Cows in the All Dairy group from Dairy A conceived to 1 of 15 different Holstein sires before lactation I and 1 of 15 Holstein sires before lactation II. Cows in the Beef on Dairy group from Dairy A conceived to 1 of 15 Holstein sires before lactation I and 1 of 3 beef breed sires before lactation II. Cows in the All Dairy group from Dairy B conceived to 1 of 199 Holstein sires before lactation I and 1 of 112 Holstein sires before lactation II. Cows in the Beef on Dairy group from Dairy B conceived to 1 of 210 Holstein sires before lactation I and 1 of 4 Simmental sires before lactation II. Both dairies used semen from the same Simmental sire for some conceptions. This was, in part, because the same genetics supply company served both dairies and provided guidance on sire selection. Season and year of conceptions before each lactation are given in Table 2.

**Table 1.** Characteristics of paired^1^ dairy cows conceived to Holstein sires (All Dairy) or a Holstein sire then beef breed sire (Beef on Dairy) in two successive lactations from two dairies

	Dairy A		Dairy B	
Item	All Dairy	Beef on Dairy	All Dairy	Beef on Dairy
Number of cows	72	72	456	456
Chronological lactation number				
2–3	30	30	258	258
3–4	27	27	97	97
4–5	15	15	54	54
5–6	–	–	37	37
6–7	–	–	9	9
7–8	–	–	1	1
Average cow age at conception for lactation I, years	2.99	2.98	3.04	3.11
Average cow age at conception for lactation II, years	3.98	3.98	4.12	4.18
Cow breed				
Holstein	72	72	431	431
Holstein × Jersey crossbred	–	–	25	25
Previous sire of conception for lactation I				
Holstein	72	72	456	456
Previous sire of conception for lactation II				
Holstein (individual sires combined)	72	–	456	–
Angus, sire A	–	7	–	–
Simmental, sire A	–	40	–	369
Simmental, sire B	–	–	–	34
Simmental, sire C	–	–	–	44
Simmental, sire D	–	–	–	9
Crossbred, sire A	–	25	–	–

Cows in the All Dairy group were matched with cows in the Beef on Dairy group based on the following criteria: same dairy, same lactation number, same cow breed, and same season and year of previous conception.

**Table 2.** Season^1^ and year of conception for paired^2^ dairy cows (Dairy A: *n* = 72/ group; Dairy B: *n* = 456/group) conceived to Holstein sires (All Dairy) or a Holstein sire then beef breed sire (Beef on Dairy) in two successive lactations from two dairies

	Conception before lactation I				Conception before lactation II			
Item	Spring	Summer	Fall	Winter	Spring	Summer	Fall	Winter
Dairy A								
2016	–	–	10	4	–	–	–	–
2017	86	–	–	44	–	–	–	–
2018	–	–	–	–	60	–	–	84
Dairy B								
2014	70	66	76	66	–	–	–	–
2015	48	64	108	76	58	60	80	56
2016	68	72	52	78	46	68	96	82
2017	24	2	–	42	66	76	58	86
2018	–	–	–	–	30	2	–	48

Spring (March to May), summer (June to August), fall (September to November), and winter (December to February).

Cows in the All Dairy group were matched with cows in the Beef on Dairy group on criteria: same dairy, same lactation number, same cow breed, and same season and year of previous conception.

Comparisons of dairy cow performance between the All Dairy and Beef on Dairy groups were evaluated from three perspectives. Firstly, comparison of groups in lactation I identified inherent differences between cow groups, regardless of sire type in previous conception because both groups were conceived to Holstein. Secondly, comparison of groups in lactation II identified differences between cow groups with different sire types of previous conception (Holstein vs. beef breed). Finally, comparison of groups for the difference between lactation I and II identified if sire type of previous conception (same for both groups in lactation I but different between groups in lactation II) affected performance in successive lactations of the same cow. Of these three perspectives, the latter controlled the most variation because, within each cow group, the same cow was evaluated in both lactations. Yet, independent comparisons of each lactation provided insight to a measure’s absolute value, which was more industry relevant.

Cow served as the experimental unit (Dairy A: *n* = 72 per group; Dairy B: *n* = 456 per group) for analysis. A two-sample *t*-test was used to test for differences in performance measures. Binary data (e.g., calf sex and stillborn incidence) were tested using a chi-square two-sample test for equality of proportions with continuity correction. Significance was defined as *P* ≤0.05, and tendencies were established as 0.05 < *P* ≤ 0.10. Sire type of previous conception was considered most influential when 1) a difference (*P* < 0.05) existed between groups for lactation II but not (*P* > 0.10) lactation I, and 2) the same difference (*P* < 0.05) existed between groups for the difference between lactations.

### Feedlot Performance

Closeout data were collected from pens of conventional beef cattle and beef × dairy crossbreds fed at a commercial feedlot in southwest Kansas from 2015 to 2018. Breed composition of conventional beef cattle varied but was representative for the feedlot and primarily consisted of Angus. In comparing cattle types, conventional beef will be referenced as “beef” throughout this manuscript for brevity. Beef × dairy crossbreds were progeny of Simmental, Angus, or Simmental × Angus bulls and Holstein cows. Data represented pen averages and consisted of animal count, body weight (BW) at arrival (unshrunk), BW at harvest (shrunk 4%), days on feed, average daily gain, gain-to-feed, dressing percentage, percentage U.S. Department of Agriculture (USDA) Choice or better, percentage USDA Yield Grade 1 to 5, and percentage dark cutters. Data were processed and analyzed using R statistical software (R Core Team, 2021). The MatchIt (Ho et al., 2011) and optmatch (Hansen and Klopfer, 2006) packages were used to select control pens (beef) that matched treatment pens (beef × dairy) in a 1:1 ratio, without replacement, according to criteria: same sex (steer vs. heifer), arrival BW not more than 45 kg different, and harvest date not more than 30 d different. By using arrival BW and harvest date as selection criteria, pens were fed similar diets during a similar timeframe to reduce influence of diet and weather but growth, to an extent, was allowed to vary. Specific diet information was not available because of the lapse in time from which lots were selected. Generally, cattle were finished on a high-concentrate diet, received two administrations of hormonal implants, and were fed recommended levels of ractopamine hydrochloride 30 d before harvest. Age at arrival and previous management (e.g., backgrounding or calf ranch) were allowed to vary between beef and beef × dairy pens. Although these factors have known influence on performance, they largely represented common industry practices, which are frequently different between the two cattle types. Data matching selection criteria were returned for 26 pens per group, where each group was comprised of 14 pens of steers and 12 pens of heifers. Data from pens of steers and heifers were combined to provide power in testing for the effect of cattle type; effect of sex was not a study objective. Pen (*n* = 26 per group) served as the experimental unit, and a two-sample *t*-test was used to test for differences between groups. Significance was defined as *P* ≤0.05, and tendencies were established as 0.05 < *P* ≤ 0.10.

### Carcass Performance

Individual carcass data for beef, beef × dairy, and Holstein cattle obtained using a VBG 2000 camera (VBG 2000; e+v Technology GmbH & Co.KG, Oranienburg, Germany) were sourced at three federally inspected, commercial beef processing facilities from harvest lots subsampled for a separate unpublished study. Cattle were harvested during the fall of 2019. Each carcass was measured for traits: hot carcass weight (HCW); fat trimmed at harvest as a percentage of HCW (included kidney, pelvic, and heart fat); video image analysis (VIA) 12th rib fat thickness; VIA ribeye area; VIA yield grade; VIA marbling score; and Angus phenotype designation (determined by processing facility personnel). All carcasses were “A” maturity and less than 30 mo of age as determined by dentition. Yield grade was calculated from the VIA system according to USDA (2017) and utilized a standardized percentage of kidney, pelvic, and heart fat of 2.5. Quality grade was determined from VIA marbling score using USDA quality grade standards (USDA, 2017), where: No Roll = 200 to 299, Select = 300 to 399, Low Choice = 400 to 499, Upper 2/3 Choice = 500 to 699, and Prime = 700 and greater. Cattle procurement leadership for the processing facilities provided verification of cattle type and sex within harvest lots, and this identity was assumed for individual carcasses. Breed composition of carcasses, which was determined through genotype testing in a separate study, was representative of common industry breeding practices at the time. Carcasses from beef cattle represented common U.S. beef breeds and (or) crossbreds of Angus, Charolais, Simmental, Limousin, and Hereford. Carcasses of beef × dairy crossbreds were comprised of 50% Angus, Simmental, Limousin, or any of their crossbred combinations and 50% Holstein or Holstein × Jersey. Holstein carcasses were confirmed for their Holstein composition.

Only data from carcasses of steers with a HCW of 373 to 418 kg were analyzed. This HCW range was selected around the median HCW of beef × dairy carcasses to maximize sample size in this group of interest, minimize variation in HCW between groups, and represent a reasonable industry average HCW (Boykin et al., 2017). Only steer carcasses were evaluated because data were not available for carcasses of Holstein heifers. Data collection characteristics for beef (*n* = 966), beef × dairy (*n* = 518), and Holstein (*n* = 935) steers matching selection criteria are displayed in Table 3.

**Table 3.** Sample collection characteristics of carcasses from conventional beef, beef × dairy, and Holstein steers

Item	Beef	Beef × dairy	Holstein
Number of carcasses	966	518	935
Number of lots sampled	24	23	19
Harvest plant location			
Kansas	135	86	58
Texas	704	419	773
Nebraska	127	13	104
Feedlot location			
Colorado	13	0	0
Iowa	66	0	104
Kansas	164	56	58
Nebraska	19	30	0
South Dakota	0	13	0
Texas	704	419	773

A linear model for each carcass characteristic was fit with breed type (beef, beef × dairy, or Holstein) as the fixed effect in R statistical software (R Core Team, 2013). Effect of breed type was evaluated using an analysis of variance. When effect of breed type was significant, means were separated with Tukey adjusted pairwise comparisons using the emmeans (Lenth, 2018) and multcomp (Hothorn et al., 2008) packages. Frequency data for VIA yield grade and USDA quality grade were tested between groups using a chi-square three-sample test for equality of proportions with continuity correction. Significance for effect of breed type and pairwise comparisons was defined as *P* ≤0.05, and tendencies for effect of breed type were established as 0.05 < *P* ≤ 0.10.

## RESULTS

### Dairy Cow Performance

At Dairy A, sire type of previous conception (Holstein vs. beef breed) produced few meaningful differences in dairy cow performance (Table 4). Gestation length and days dry before freshening were the only traits not different (*P* ≥ 0.33) at lactation I, different (*P* ≤ 0.05) at lactation II, and different (*P* ≤ 0.03) between lactations. Gestation length was 3 d greater (*P* < 0.01) in cows conceived to beef sires (Beef on Dairy) compared to Holstein sires (All Dairy) at lactation II, and the difference in gestation length between lactations was 3 d greater (*P* < 0.01) for Beef on Dairy. As a somewhat indirect consequence of these gestation length differences, the difference between lactations in calving interval tended to be 15 d greater (*P* = 0.07) for Beef on Dairy. Other measures of cow performance at Dairy A were not influenced (*P* > 0.10) by sire type of previous conception.

**Table 4.** For Dairy A, paired comparison^1^ of performance measures and their difference (DIFF) between two successive lactations (LACT I and II) for dairy cows (*n* = 72/group) previously conceived to Holstein sires (All Dairy) or a Holstein sire then beef breed sire (Beef on Dairy)

	All Dairy			Beef on Dairy			LACT I SEM^2^	LACT II SEM^2^	LACT DIFF SEM^2^	LACT I *P*-value	LACT II *P*-value	LACT DIFF *P*-value
	LACT I	LACT II	LACT DIFF	LACT I	LACT II	LACT DIFF						
Item	(Dairy)	(Dairy)		(Dairy)	(Beef)							
Reproduction (previous lactation)												
Days open	92	86	−7	87	93	5	4.5	5.1	5.8	0.45	0.31	0.15
Times bred	2.1	1.9	−0.2	1.8	2.1	0.2	0.17	0.18	0.2	0.24	0.57	0.15
First service conceptions, %	47	57		53	46		3.7	3.8		0.62	0.24	
First and second service conceptions, %	72	78		79	81		4.1	4.1		0.44	0.84	
Gestation length, days	276	276	1	275	279	4	0.8	0.5	0.9	0.83	<0.01	<0.01
Calving interval, days	368	362	−6	363	372	9	4.7	5.2	5.8	0.45	0.15	0.07
Reproduction (current lactation)												
Days in milk at first breeding	60	66	6	64	66	2	1.3	1.5	1.7	0.05	0.80	0.08
Days in milk at first heat	50	66	16	56	66	10	1.8	1.5	2.1	0.04	0.80	0.05
Days open	86	74	−12	93	75	−18	5.1	1.9	5.6	0.31	0.73	0.15
Lactation												
Total milk, kg	11,590	11,770	180	12,000	11,940	−60	335	211	254	0.34	0.56	0.49
Days in milk	306	294	−12	314	294	−19	5.0	2.0	5.3	0.27	0.91	0.27
ADM (average daily milk), kg/day	38	40	2	38	41	2	0.9	0.7	0.8	0.83	0.53	0.72
305-d MHE^3^, kg	13,040	12,460	−580	13,170	12,380	−790	332	216	240	0.76	0.81	0.54
Peak daily milk, kg	51	52	1	51	53	2	1.2	0.9	1.1	0.93	0.36	0.55
Days in milk at peak	85	65	−20	83	57	−25	8.4	3.8	8.9	0.81	0.08	0.64
ADM in week 4, kg/day	45	48	3	46	49	4	1.2	1.0	1.3	0.57	0.24	0.76
Days dry before freshening	53	56	2	52	58	6	0.9	1.0	1.4	0.33	0.05	0.03
Calf characteristics												
Stillbirth, %	1	0		1	4		0.7	1.2		1.00	0.24	
Twinning, %	1	8		3	6		1.0	1.7		1.00	0.74	
Males, %	64	58		53	53		3.9	3.8		0.24	0.61	
Cow health												
Laminitis, %	4	1		7	7		1.5	1.5		0.72	0.21	
Mastitis, %	11	26		17	35		2.3	3.2		0.47	0.37	

Cows in the All Dairy group were matched with cows in the Beef on Dairy group on criteria: same dairy, same lactation number, same cow breed, and same season and year of previous conception.

Largest standard error of the means (SEM).

MHE, mature herd equivalent.

At Dairy B, few differences were related to sire type of previous conception, and lactation performance indicated selection pressure from management to breed more productive cows to Holstein versus beef sires (Table 5). Gestation length was not different (*P* = 0.74) between groups at lactation I but was 2 d greater (*P* < 0.01) for Beef on Dairy at lactation II. The difference between lactations in gestation length was 1 d greater (*P* < 0.01) for Beef on Dairy. This greater gestation length did not seem to impact calving interval, which was not different (*P* = 0.91) between groups at lactation II. Beef on Dairy exhibited a 6 d greater (*P* = 0.05) calving interval and 7 greater (*P* = 0.05) days open before lactation I. This might suggest that, before lactation I, cows in the Beef on Dairy group were more difficult to breed, although number of times bred was not different (*P* = 0.11). This result may have contributed to cows in the Beef on Dairy group exhibiting 2 greater (*P* ≤ 0.02) days before freshening at lactation I and lactation II. From lactation I to II, cows in the Beef on Dairy group experienced 8 fewer (*P* = 0.05) days open and tended to require 0.3 fewer (*P* = 0.06) times bred before conception. During both lactations, Beef on Dairy exhibited less (*P* < 0.01) total milk yield (by up to 1,320 kg), average daily milk yield (by up to 4 kg), 305-d MHE (by up to 1,380 kg), and peak daily milk yield (by up to 6 kg). However, the difference between lactations for these same measures was greater (*P* ≤ 0.03) for Beef on Dairy. This suggested that while cows in the Beef on Dairy group were lower producing, milk yield was not affected by sire type of previous conception. A lower potential for milk yield could be hypothesized as an explanation for the lesser (*P* = 0.01) incidence (by 6 percentage units) of mastitis for Beef on Dairy in lactation II. All other measures of cow performance at Dairy B were not influenced (*P* > 0.10) by sire type of previous conception.

**Table 5.** For Dairy B, paired comparison^1^ of performance measures and their difference (DIFF) between two successive lactations (LACT I and II) for dairy cows (*n* = 456/group) previously conceived to Holstein sires (All Dairy) or a Holstein sire then beef breed sire (Beef on Dairy)

	All Dairy			Beef on Dairy			LACT I SEM^2^	LACT II SEM^2^	LACT DIFF SEM^2^	LACT I *P*-value	LACT II *P*-value	LACT DIFF *P*-value
	LACT I	LACT II	LACT DIFF	LACT I	LACT II	LACT DIFF						
Item	(Dairy)	(Dairy)		(Dairy)	(Beef)							
Reproduction (previous lactation)												
Days open	113	115	2	120	114	-6	2.4	1.7	2.9	0.05	0.56	0.05
Times bred	2.0	1.9	0.0	2.1	1.9	-0.3	0.06	0.06	0.08	0.11	0.35	0.06
First service conceptions, %	48	49		44	51		1.4	1.4		0.29	0.64	
First and second service conceptions, %	73	75		72	76		1.6	1.6		0.82	0.70	
Gestation length, days	277	277	1	277	279	2	0.3	0.3	0.3	0.74	<0.01	<0.01
Calving interval, days	390	392	2	396	393	-4	2.5	1.7	2.9	0.05	0.91	0.12
Reproduction (current lactation)												
Days in milk at first breeding	88	88	0	88	88	0	0.3	0.4	0.5	0.32	0.34	0.17
Days open	115	124	9	114	120	6	1.8	2.0	2.6	0.77	0.14	0.34
Lactation												
Total milk, kg	13,770	14,330	560	12,450	13,380	930	103	103	120	<0.01	<0.01	0.03
Days in milk	337	344	7	336	341	5	1.7	1.9	2.4	0.52	0.17	0.52
Average daily milk, kg/day	41	42	1	37	39	2	0.2	0.2	0.3	<0.01	<0.01	<0.01
305-d MHE^3^, kg	13,130	12,670	-460	11,750	11,870	120	85	77	90	<0.01	<0.01	<0.01
Peak daily milk, kg	54	55	2	48	52	4	0.3	0.4	0.4	<0.01	<0.01	<0.01
Days in milk at peak	62	64	2	62	60	-2	1.9	1.8	2.5	0.81	0.21	0.27
Days dry before freshening	49	55	5	51	57	6	0.5	0.4	0.6	0.02	<0.01	0.71
Calf characteristics												
Stillbirth, %	1	2		2	3		0.3	0.4		0.79	0.35	
Twinning, %	2	5		3	3		0.4	0.5		0.55	0.12	
Males, %	46	51		51	54		1.4	1.5		0.15	0.51	
Cow health												
Laminitis, %	34	42		32	39		1.2	1.3		0.67	0.42	
Mastitis, %	16	19		13	13		0.9	1.0		0.30	0.01	

Cows in the All Dairy group were matched with cows in the Beef on Dairy group on criteria: same dairy, same lactation number, same cow breed, and same season and year of previous conception.

Largest standard error of the means (SEM).

MHE, mature herd equivalent.

### Feedlot Performance

Compared to beef steers and heifers, beef × dairy steers and heifers were generally less efficient in the feedlot but produced a greater proportion of carcasses with a lower yield grade (Table 6). At average daily gains and harvest weights that were not different (*P* ≥ 0.22), beef × dairy crossbreds tended to have 5% lesser (*P* = 0.07) feed conversion than beef cattle. Beef × dairy crossbreds also converted less BW into HCW, with a 1 percentage unit lesser (*P* < 0.01) dressing percentage. Yet, at the carcass level, beef × dairy crossbreds produced a greater (*P* < 0.01) percentage of yield grade 2 carcasses and a lesser (*P* ≤ 0.01) percentage of yield grade 4 and 5 carcasses. Cattle of both types produced approximately 80% Choice or better carcasses and did not differ (*P* = 0.67) for this metric of grading percentage.

**Table 6.** Paired comparison^1^ of feedlot closeout characteristics for conventional beef and beef × dairy cattle in the same feedlot

Item	Beef	Beef × dairy	SEM^2^	*P*-value
Number of pens	26	26	–	–
Total animal count	1,536	1,551	–	–
Body weight at arrival, kg	362	366	9.2	0.74
Body weight at harvest, kg	603	617	9.3	0.22
Days on feed	159	167	5.2	0.24
Average daily gain, kg/d	1.53	1.51	0.042	0.24
Gain:feed	0.149	0.142	0.0029	0.07
Dressing percentage	64.2	63.2	0.18	<0.01
USDA Choice or better, %	79.3	81.1	3.30	0.67
USDA yield grade, %				
Yield grade 1	6.6	8.1	1.41	0.43
Yield grade 2	36.5	47.0	2.52	<0.01
Yield grade 3	40.4	38.3	2.13	0.49
Yield grade 4	15.3	6.5	2.33	<0.01
Yield grade 5	1.0	0.1	0.31	0.01
Dark cutters, %	0.7	0.7	0.54	0.96

Pens were paired according to same sex, initial body weight not more than 45 kg different, and harvest date not more than 30 d different.

Largest standard error of the means (SEM).

### Carcass Performance

Generally, carcasses from beef × dairy steers were leaner than beef steers and heavier muscled than Holstein steers (Table 7). Although carcasses were selected within a 45 kg range of HCW, beef steers had greater (*P* < 0.05) HCW than beef × dairy and Holstein steers, but only by 3 and 4 kg, respectively. Beef × dairy cattle possessed 18% lesser (*P* < 0.05) 12th rib fat than beef cattle and 5% greater (*P* < 0.05) ribeye area than dairy cattle. Despite differences in HCW, 12th rib fat, and ribeye area, mean yield grade—although statistically different—was not more than 0.1 units different between cattle types. However, yield grade was calculated using a standardized kidney, pelvic, and heart fat percentage across all cattle types. Actual amount of kidney, pelvic, and heart fat, alongside other hot fat trimming, was included in fat trimmed at harvest. Beef × dairy and Holstein carcasses had an almost 1-unit greater (*P* < 0.01) percentage of fat trimmed at harvest than beef steers. Beef × dairy and Holstein cattle were not different (*P* > 0.10) in their quality or yield grade distributions. Mean marbling score was more than 30 degrees lesser (*P* < 0.05) in beef carcasses than beef × dairy and Holstein carcasses. At a compromise to upper 2/3 Choice and low Choice, beef cattle demonstrated a quality grade distribution shifted towards a greater (*P* < 0.05) proportion of Select, by up to 25 percentage units, compared to beef × dairy and Holstein cattle. Additionally, beef cattle produced nearly double (*P* < 0.05) the percentage of yield grade 4 carcasses produced by beef × dairy and Holstein cattle.

**Table 7.** Characteristics of 373–418 kg carcasses from conventional beef, beef × dairy, and Holstein steers captured from video image analysis (VIA)

Item	Beef	Beef × dairy	Holstein	SEM^1^	*P*-value
Number of carcasses	966	518	935	–	–
Hot carcass weight (HCW), kg	397^a^	394^b^	393^b^	0.6	<0.01
12th rib fat thickness, cm	1.31^a^	1.11^b^	0.92^c^	0.018	<0.01
Ribeye area, cm^2^	94.8^a^	92.2^b^	87.5^c^	0.43	<0.01
Fat trimmed at harvest^2^, % of HCW	3.56^b^	4.51^a^	4.55^a^	0.043	<0.01
VIA yield grade^3^	2.92^a^	2.82^b^	2.86^ab^	0.031	0.02
Marbling score	447^b^	481^a^	482^a^	4.7	<0.01
VIA yield grade, % of total					
Yield grade 1	12.0^a^	8.1^ab^	7.6^b^	1.20	<0.01
Yield grade 2	42.9^b^	56.6^a^	52.0^a^	2.18	<0.01
Yield grade 3	35.6	31.3	36.8	2.04	0.10
Yield grade 4	8.4^a^	3.9^b^	3.4^b^	0.89	<0.01
Yield grade 5	1.1^a^	0.2^a^	0.2^a^	0.34	0.01
Quality grade^4^, % of total					
Prime	3.7	4.1	4.3	0.87	0.83
Upper 2/3 Choice	20.1^b^	30.9^a^	31.7^a^	2.03	<0.01
Low Choice	28.8^b^	42.5^a^	39.8^a^	2.17	<0.01
Select	47.3^a^	22.4^b^	24.2^b^	1.83	<0.01
No Roll	0.1	0.2	0.1	0.19	0.88
Angus phenotype^5^, %	58^b^	78^a^	0^c^	1.7	<0.01

Largest standard error of the means (SEM).

Fat trimmed at harvest includes kidney, pelvic, and heart and additional hot fat trimming.

Calculated using a standard kidney, pelvic, and heart fat percentage of 2.5.

Quality grade was determined from VIA marbling score using standards (USDA, 2017), where: No Roll = 200–299, Select = 300–399, Low Choice = 400–499, Upper 2/3 Choice = 500–699, and Prime = 700 and greater.

Determined by plant personnel for eligibility into Angus-specific branded programs.

Means in the same row lacking a common superscript differ (*P* < 0.05).

## DISCUSSION

For dairymen, the most meaningful conclusion of this work was that breeding beef sires to dairy cows had minimal impact on cow performance traits related to profitability, like milk yield and days in milk. Only gestation length contributed to slight differences between the breeding schemes studied, a result that was repeated across two independent operations using different cows. Dairy producers may need to account for a 2 to 3 d greater gestation length in managing late prepartum dairy cows conceived to beef sires.

Scanavez and Mendonça (2018) reported dairy cows conceived to Angus sires had a 1.6 d greater gestation length than cows conceived to Holstein sires. Sires of multiple other beef breeds, including Simmental, Hereford, Charolais, Limousine, and Belgian Blue, have also shown greater gestation length when mated to dairy cows compared to Holstein sires (Badi et al., 1985; O’Ferrall and Ryan, 1990; Fouz et al., 2013). Scanavez and Mendonça (2018) suggested that greater gestation length might result from the smaller size of Angus-sired calves compared to Holstein-sired calves. Although, Badi et al. (1985) reported Charolais- and Simmental-sired calves shared heavier birth weights and greater gestation length than Friesian-sired calves. Other variables known to affect gestation length, including lactation number, season of calving, cow breed, cow age, twinning, and offspring sex (Badi et al., 1985; Norman et al., 2009; Scanavez and Mendonça, 2018), were either reasonably controlled or not different between groups in this study. Norman et al. (2009) demonstrated an effect of individual sire on gestation length within dairy breeds, suggesting that individual sire may have just as much of an effect as breed or breed type alone.

Differences in gestation length were not concomitant with incidence of stillbirths in this study. Fouz et al. (2013) reported that greater gestation length did not result in greater calving difficulty. Calving difficulty and calf birthweights were not available here. However, data management could have artificially removed cases of calving difficulties. For example, only complete lactation records were considered, where cows that died from a difficult calving were excluded. Additional research is needed to understand biological mechanisms behind gestation length and calving difficulty that might be influenced by sire type of conception.

The difference between lactations in milk yield that was more desirable for Beef on Dairy at Dairy B was unexpected, and it was not an observation noted at Dairy A. Higher-producing cows in the All Dairy group at Dairy B might have experienced greater difficulty maintaining consistently high milk production across successive lactations. Nonetheless, subsequent milk yields were not suggested to improve by breeding dairy cows to beef sires. Rather, milk yields were not influenced by breeding dairy cows to beef sires, which has been supported by others (Badi et al., 1985; O’Ferrall and Ryan, 1990; Scanavez and Mendonça, 2018).

Relatively few individual beef sires were represented in the Beef on Dairy group compared to the much greater number of Holstein sires of the All Dairy group. Results for the Beef on Dairy group in this study may be influenced by these individual sires to a greater degree than the overall population of beef sires. Moreover, the number of beef breeds represented between these sires was limited. McWhorter et al. (2020) and Pereira et al. (2022) demonstrated that, in U.S. beef × dairy crossbreeding systems, Angus was the predominant sire breed of choice. Compared to these studies, Halfman and Sterry (2019) reported a lesser percentage use of Angus (although it was still predominant) and a greater percentage of other breeds, including Simmental, Limousin, and their Angus-influenced composites. Here, Simmental sires represented a greater proportion of conceptions in cow performance data than what they likely represent in total U.S. beef × dairy conceptions. Both dairies evaluated in this study selected individual beef sires under the direction of a bull stud. Hence, some of the reported challenges associated with beef sires may have been negated by this guidance.

When steers and heifers of beef and beef × dairy types were placed at similar weights and fed during similar timeframes in the same feedlot, growth performance of these cattle was not vastly different. The lower feed efficiency in beef × dairy crossbreds could be attributed to the influence of dairy genetics that require greater energy for maintenance than beef genetics (Garrett, 1971). Subsequently, a lower conversion of feed energy into carcass tissue (protein and fat) could be attributed to the lower dressing percentage associated with beef × dairy crossbreds (Garrett, 1971). Results of this study should be interpreted under the context that, because of the observational nature and retrospective collection of feedlot closeout data, very little could be done to control for factors known to influence feedlot performance, like genetics and management before feedlot entry. However, these factors were thought to be representative of standard industry practices for the cattle types. To provide a more powerful test of cattle type in hopes of overcoming some of these logistical limitations, feedlot closeout data from steers and heifers were combined and results should be interpreted as such.

This study sampled carcasses at relatively similar HCW from a multitude of beef processing facilities, harvest lots, and geographical region. These survey-like data indicated that, at harvest, beef × dairy crossbreds may realize an optimum balance of external leanness associated with dairy cattle and muscling associated with beef cattle without compromise to carcass quality. These results, however, were derived from USDA yield grade associated traits, which have been suspected for their ineffectiveness in predicting carcass red meat yield, particularly in dairy cattle (Lawrence et al., 2010). Fat trimmed at harvest, if indicative of kidney, pelvic, and heart fat, may be an important factor of consideration when evaluating carcass yield of beef × dairy crossbreds. Greater deposition of carcass fat towards in internal depots, and away from subcutaneous depots, in dairy cattle compared to beef cattle has been previously reported (Callow, 1961; Kempster et al., 1976; Dolezal et al., 1993). The authors suggested that these differences in fat partitioning might be a product of divergent selection for milk and meat characters in different cattle types.

Straightbred dairy cattle have traditionally comprised a considerable proportion of the Prime grade (Boykin et al., 2017). Beef × dairy crossbreds in this study were not greatly different from Holsteins in quality grade distribution, although a much larger sample size is likely needed to fully understand how an industry shift from slaughtering Holsteins to beef × dairy crossbreds will affect the national Prime percentage. In this study, the quality grade distribution of conventional beef carcasses was not entirely representative of the U.S. average. The most recent National Beef Quality Audit reported that 24% of carcasses graded Select, whereas nearly double that (47%) of beef carcasses in this study graded Select (Boykin et al., 2017). The quality grade distributions of beef × dairy and Holstein carcasses more closely aligned with the national averages of Boykin et al. (2017). However, a large proportion of beef, beef × dairy, and Holstein carcasses evaluated here were produced from cattle fed in Texas, which traditionally exhibits a quality grade distribution shifted more towards Select compared to the national average. Hence, like Holsteins, beef × dairy crossbreds may positively contribute to the national average quality grade distribution.

Results from this study should be perceived with an understanding of the timeframe in which measurements were collected. Feedlot closeout data were collected between 2015 and 2018, and carcass data were obtained from cattle slaughtered in 2019. Since these times, beef × dairy crossbreeding has become a much more widespread practice among U.S. dairies according to recent reports (National Association of Animal Breeders [NAAB], 2021; Baisel and Felix, 2022). Demand from dairies for beef semen from genetics supply companies has prompted considerable improvement in the type of beef genetics being supplied to produce beef × dairy crossbreds (Baisel and Felix, 2022). Many genetics supply companies have recently developed specific branded programs for genetics of beef sires best suited for producing crossbreds that optimize performance and profitability in the beef supply chain. Here, results do not reflect these recent and rapid improvements in genetics. It could only be speculated that feedlot and carcass performance of beef × dairy crossbreds in today’s system would be improved to the values reported in this study. Still, this study filled a void in performance data of U.S. crossbreds that is largely lacking in the literature.
